# Effects of Rosemary Oil (*Rosmarinus officinalis*) On The Shelf-Life of Minced Rainbow Trout (*Oncorhynchus mykiss*) during Refrigerated Storage 

**DOI:** 10.3390/foods1010028

**Published:** 2012-12-04

**Authors:** Pier Giorgio Peiretti, Francesco Gai, Marco Ortoffi, Riccardo Aigotti, Claudio Medana

**Affiliations:** 1 Institute of Science of Food Production, National Research Council, Via L. da Vinci, 44, 10095 Grugliasco, Italy; E-Mails: francesco.gai@ispa.cnr.it (F.G.); marco.ortoffi@ispa.cnr.it (M.O.); 2 Department of Molecular Biotechnology and Health Sciences, University of Torino, Via P. Giuria, 5, 10125 Torino, Italy; E-Mails: riccardo.aigotti@unito.it (R.A.); claudio.medana@unito.it (C.M.)

**Keywords:** rainbow trout, rosemary oil, oxidative stability, fatty acid, biogenic amine, terpene

## Abstract

The effects of three concentrations (0.2%, 1% and 3%) of rosemary oil (RO) on the freshness indicators, oxidative stability, fatty acid and biogenic amine (BA) contents of minced rainbow trout muscle (MTM) were investigated after different periods of storage (three and nine days) at 4 ± 1 °C. Moreover, the terpene and sesquiterpene contents in the treated MTM were also measured. RO treatment improves the pH, oxidative stability of the lipids and the FA profile, which resulted in a significant extension of MTM shelf-life. Storage time influenced all freshness indicators, with the exception of yellowness and chroma. Treatment with RO had a positive effect, leading to low BA content, especially putrescine, cadaverine, tyramine and histamine. Differences in BA were also found to be due to storage time, with the exception of spermidine, which was not influenced by time. Moreover, the presence of the terpenoid fraction of RO in MTM improved the quality of this ready-to-cook fish food.

## 1. Introduction

The food industry has offered ready-to-eat and ready-to-cook products for people who have little time for eating, and therefore it is very important to increase product shelf-life by retarding food quality degradation. Consequently, there has been a growing interest in the use of natural substances with antioxidant properties to replace traditional synthetic additives (BHT, BHA and TBHQ), whose carcinogenic activity has been demonstrated [1]. 

The scientific literature on food preservation techniques to improve the oxidative stability of fish-based foods reports renewed research interest in natural compounds of vegetable origin as valid alternatives to synthetic preservatives. Herbs and spices are traditionally used as food ingredients, as well as for their antioxidant properties. Natural antioxidants are currently receiving considerable attention in human nutrition and good starting material is provided by secondary metabolites, such as the polyphenolic compounds present in plants that have been reported to have antioxidant properties [2]. 

Extract of rosemary (*Rosmarinus officinalis*), whose antioxidant activity is well known [3,4], is the most abundantly tested active natural agent in both food-simulating systems and in real food matrices [5,6,7]. The antioxidative properties of rosemary are mainly related to their content of phenolic compounds, suggesting that their antioxidant actions are similar to those of synthetic phenolic antioxidants [8]. The antioxidant capacity of rosemary is attributed to three phenolic diterpenoids (carnosic acid, carnosol, rosemaric acid) [9], but many other components (rosmanol, epirosmanol, isorosmanol, rosmaridiphenol, rosmadial, rosmariquinone, carvacrol, carvone, cymene, cineole, fenchone, limonene, terpinene and thymol) are expected to contribute to its antioxidative and antimicrobial properties [10]. Murphy *et al*. [11] investigated the antioxidant properties of rosemary oleoresin extract in pre-cooked roast beef slices during refrigerated (+3 °C) and frozen (−20 °C) storage. They reported that the addition of rosemary in the presence of salt, a pro-oxidant, resulted in lower thiobarbituric-acid reactive substance (TBARS) values during refrigerated storage, but not during frozen storage, compared with the control. 

Fresh fish is a highly perishable product, due to its biochemical composition [12]. The main cause of deterioration is the activity of typical spoilage microorganisms, provoking loss of essential fatty acids, fat-soluble vitamins and protein functionality, production of biogenic amines (BAs), and formation of off-odors [13]. 

There is little conclusive information on the effects of RO on lipid oxidation and BAs in fish flesh treated with this natural antioxidant. There are only two studies, one on the effect of rosemary and sage tea extract on BA production in *Sardina pilchardus* fillets [14], and the other on the effect of icing with rosemary extract on the oxidative stability [15] and BA formation in *Sardinella aurita* during chilled storage [16]. The objective of this investigation was to study the effects of RO addition on the shelf-life of minced rainbow trout (*Oncorhynchus mykiss*) during refrigerated storage.

## 2. Experimental Section

### 2.1. Preparation of Minced Fish Muscle Samples

Minced trout muscle (MTM) was prepared from four ice-stored rainbow trout (average weight: 657 ± 97 g) bought in a local supermarket (arrival time of fish after harvest: 24 h). Fish were filleted and after removing the skin and the bones, the muscle was ground through a grinder (Platone, Torino, Italy). MTM samples were shaped into patties of 11 cm diameter and 1.5 cm thickness using a glass shaper. For each group, six patty samples were produced in total. To study its dose-dependent efficacy, water-soluble rosemary oil (RO; Dr. Taffi, La California, Italy) was added separately to each MTM portion to give final concentrations of 0% (control, 5 mL distilled water/100 g MTM), 0.2% (0.2 mL RO + 4.8 mL distilled water/100 g MTM), 1% (1 mL RO + 4 mL distilled water/100 g MTM) and 3% (3 mL RO + 2 mL distilled water/100 g MTM).

In order to simulate the retail process, the patty groups were wrapped in plastic film and stored in a refrigerated room (4 ± 1 °C). After 3 and 9 days of refrigerated storage, the shelf-life of the MTM samples was evaluated using a series of physical and chemical analyses.

### 2.2. pH Measurement

The pH measurement was performed according to Bao *et al*. [17]. A portion (3 g) of MTM was homogenized with 20 mL of distilled water in a mixer (Moulinex Masterchef 22, type 920; Moulinex, France) for 30 s at maximal velocity. The homogenate was centrifuged at 10,000 rpm for 10 min at 4 °C and the supernatant was collected. The pH of the supernatant was then measured with a Crison MicropH 2001 pH meter (Crison Instruments, Barcelona, Spain).

### 2.3. TBARS Measurement

Lipid oxidation was determined on the MTM using modified thiobarbituric acid (TBA) analysis according to the iron-induced TBARS procedure described by Sárraga *et al*. [18]. The assay was performed at 30 min of incubation and absorbance was read at 532 nm. Liquid malondialdehyde bis(diethyl acetal) (MDA) (Aldrich Chemical Co. Ltd., Dorset, UK) was used as the standard to determine the linear standard response and recovery. The TBARS values were expressed as mg of MDA/kg of muscle tissue. All measurements were performed in triplicate.

### 2.4. Color Measurement

MTM color was measured using a bench colorimeter Chroma Meter CR-400 Konica Minolta Sensing (Minolta Sensing Inc, Osaka, Japan) in the CIELAB color space [19]. Its lightness (*L* *), redness (*a* *) and yellowness (*b* *) were recorded, and the chroma (C *) and hue (H^0^) indices were calculated as chroma: C * = [(*a* *)^2^ + (*b* *)^2^]^1/2^, and hue: H^0^ = tan^−1^(b */a *) [20]. Chroma is related to the quantity of pigments and high values represent a more vivid color and denote a lack of greyness. Hue is the attribute that permits colors to be classified as red, green, yellow, blue, and so on. Three readings were taken on the surface of the MTM and averaged.

### 2.5. Gas-Chromatographic Analysis of the Fatty Acids

Fatty acid (FA) composition was determined on the MTM samples. Lipid extraction of the samples was performed according to Peiretti and Meineri [21]; the extract was expressed as crude fat and used for the *trans*-methylation of the FAs. The FA methyl esters in hexane were then injected into a gas chromatograph (Dani Instruments S.P.A.GC1000 DPC; Cologno Monzese, Italy) equipped with a flame ionisation detector. The separation of the FA methyl esters was performed using a Famewax™ fused silica capillary column (30 m × 0.25 mm (i.d.), 0.25 μm) (Restek Corporation, Bellefonte, PA, USA). The peak area was measured using a Dani Data Station DDS 1000. Each peak was identified and quantified on the basis of pure methyl ester standards (Restek Corporation, Bellefonte, PA, USA). All analyses were performed in triplicate.

### 2.6. Sample Preparation for HPLC-MS/MS and GC/MS Analyses

Two extraction procedures were carried out on each MTM lyophilized sample. In order to measure BA concentration, 300 mg of freeze-dried flesh were extracted using 3 mL of 5 mM heptafluorobutanoic acid in water-methanol 1:1 v/v, centrifuged (10,000 rpm) and spiked with synephrine as internal standard (final concentration 100 µg/L). Terpenes were extracted by treating 30 mg of MTM with 3 mL of dichloromethane without internal standard. Each extraction was performed in triplicate.

### 2.7. Determination of Biogenic Amines

The chromatographic separations were run on a Varian 920-LC HPLC coupled with a triple quadrupole mass spectrometer 320-MS (Varian, Leinì, Italy) through an atmospheric pressure interface and an ESI ion source. Samples were analyzed for detecting BAs using an RP C18 column (Phenomenex Luna 150 mm × 2.1 mm, 3 μm particle size) at 200 μL/min flow rate. Gradient mobile phase composition was adopted: 95/5 to 40/60 in 25 min 5 mM heptafluorobutanoic acid/methanol. Injection volume was 20 μL. We used a procedure described in the literature [22], with slight modifications. The analyses were run using MS/MS acquisition. The tuning parameters adopted for the ESI source were as follows: source voltage 4.5 kV, capillary voltage 82 V, shield voltage 450 V, drying gas temperature 300 °C. The followed transitions were 89 to 72 *m/z* (collision energy 7.5 V) for putrescine, 103 to 86 *m/z* (collision energy 6.5 V) for cadaverine, 146 to 129 *m/z* (collision energy 8.5 V) for spermidine, 138 to 121 *m/z* (collision energy 6.5 V) for tyramine, 203 to 129 *m/z* (collision energy 8.5 V) for spermine, 112 to 95 *m/z* (collision energy 7.0 V) for histamine, 154 to 137 *m/z* (collision energy 10.5 V) for dopamine and 168 to 150 *m/z* (collision energy 5.5 V) for synephrine. The retention times of the analytes were 15.8 min (putrescine), 16.2 min (cadaverine), 22.1 min (spermidine), 15.9 min (tyramine), 24.8 min (spermine), 13.4 min (dopamine), 15.6 min (histamine) and 12.4 min (synephrine). The method showed a satisfactory linearity in the range 0.01 (LLOQ)–500 μg/g.

### 2.8. Gas-Chromatographic Analysis of Terpenes and Sesquiterpenes

**Figure 1:**
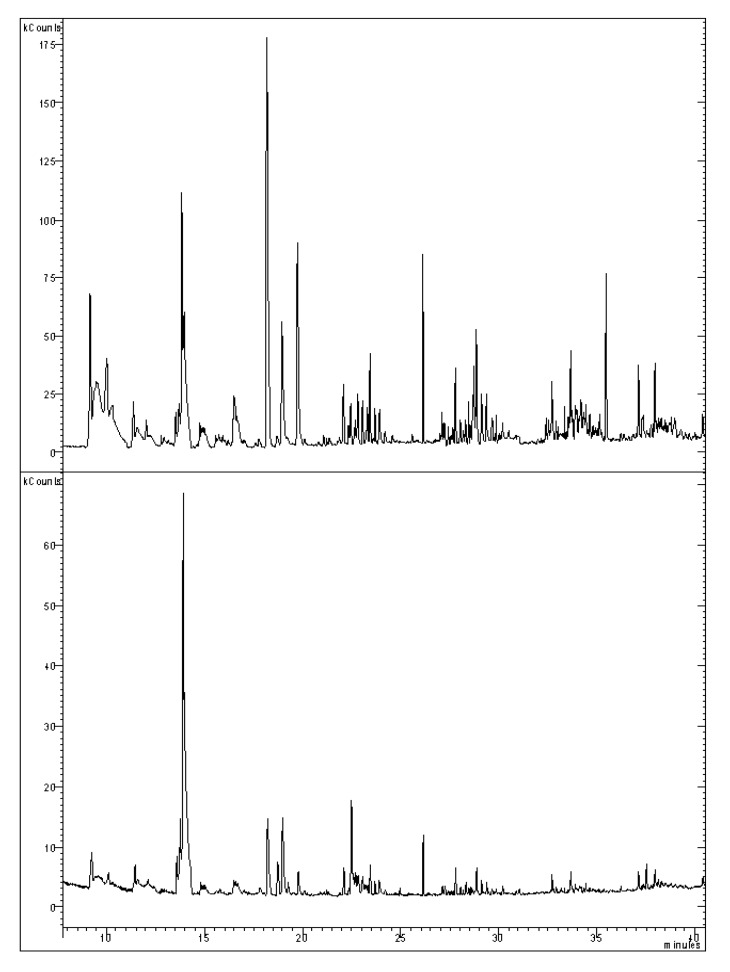
Full mass spectrometry signal obtained by GC-MS analysis of a trout extract sample (3% rosemary oil treated) (top) and of pure rosemary oil (1/100000 v/v dilution in dichloromethane) (bottom). GC-MS conditions are reported in the experimental part.

The main components of the RO added to MTM were identified by GC-MS (Varian GC-3900 with Saturn 2100T MS analyzer; Leinì, Italy). The separation was performed using a procedure previously described [23] with slight modifications: a Zebron ZB-264 fused silica capillary column (30 m × 0.25 mm (i.d.), 1.40 µm film) (Phenomenex, Torrance, CA, USA) was used. The oven temperature was initially set at 60 °C (hold time 8 min), with a gradient from 50 to 200 °C (5.0 °C/min, hold 2 min), and from 200 to 320 °C (25 °C/min, hold 1 min); injector temperature 270 °C. Column flow 1.20 mL/min. Carrier gas helium 5.5; ionization energy 70 eV; the split/splitless ratio was set to 1:10 after 45 s. Analytes were quantified by comparison with calibration curves generated by injecting increasing amounts of standard solutions obtained by dilution of a sample of the RO (Figure 1). Each peak was identified on the basis of mass spectra by matching with the components of the NIST commercial library mass spectral database (vers. 6.41). The mass-to-charge acquisition range was 40–650 *m/z*.

### 2.9. Statistical Analysis

Data were evaluated by means of the GLM procedure of the SPSS software package (version 11.5.1 for Windows, SPSS Inc., Chicago, IL, USA) and by considering the treatment, days of storage and their interaction as the main effects. The data were presented as the means of each group and the standard error of the means (SEM) together with the significance levels of the main effects and interactions. Significance was established at *p* < 0.05.

## 3. Results and Discussion

### 3.1. Freshness Indicators

The RO treatment influenced pH, TBARS and flesh color, while time influenced only pH, TBARS, L *, *a* * and hue (Table 1). The samples treated with rosemary extract were more stable to oxidation than the control sample, with the exception of flesh treated with 3.0 mL of RO, in which TBARS values increased, in particular after three days of storage.

**Table 1:** pH, thiobarbituric acid reactive substances (TBARS, as mg malondialdehyde/kg of muscle) and flesh color variables ranged according to treatment ^a^ and storage time.

	C		RO 0.2		RO 1.0		RO 3.0		SEM	Significance		
Days	3	9	3	9	3	9	3	9		Treatment	Time	Interaction
pH	6.75	7.04	6.70	7.01	6.73	6.84	6.65	6.89	0.003	0.007	0.000	0.044
TBARS	0.41	0.23	0.27	0.13	0.19	0.18	0.90	0.26	0.017	0.000	0.000	0.004
L *	70.7	71.2	70.6	75.8	75.2	76.1	80.2	79.9	0.328	0.000	0.000	0.000
a *	2.66	2.72	3.01	2.79	1.31	2.51	0.79	0.29	0.020	0.000	0.030	0.000
b *	12.1	11.1	11.6	13.4	12.6	11.9	12.2	13.2	0.120	0.000	0.075	0.000
Chroma	12.4	11.4	12.0	13.7	12.7	12.2	12.2	13.2	0.123	0.001	0.053	0.000
Hue	77.6	76.2	75.5	78.3	84.1	78.1	86.3	88.7	0.345	0.000	0.035	0.000

^a^ C: control; RO 0.2: rosemary oil 0.2%; RO 1.0: rosemary oil 1.0%; RO 3.0: rosemary oil 3.0%.

RO-treated samples enriched with 0.2 and 1.0 mL of RO had lower TBARS values than those in the control during storage. This result agrees with the reports of Akhtar *et al*. [5], who found that oxidative stability of lipids was further improved by applying rosemary oleoresin to the surface of rainbow trout fillets.

Formanek *et al*. [24] showed that in minced beef meat produced from dietary vitamin E-supplemented *semimembranosus* muscles, oxidative stability was improved by adding antioxidants under aerobic packaging conditions or under MAP packaging containing elevated oxygen levels, during refrigerated and illuminated display at 4 °C for eight days. They reported that rosemary extract was as effective in reducing TBARS as the combination of synthetic antioxidants, BHA/BHT. Tironi *et al*. [25] evaluated the effect of the application of rosemary extract (200 and 500 mg/kg), and determined the lipid and protein alterations in minced Argentine seaperch (*Pseudopercis semifasciata*) muscles during frozen storage (−11 °C). Lipid oxidation reached maximum TBARS values between three and four months of storage in untreated muscles. They found that rosemary extract reduced lipid oxidation in three-month (200 mg/kg) or six-month (500 mg/kg) stored muscles.

Tironi *et al*. [26] found that the application of 200 or 500 mg/kg rosemary extract significantly reduced production of secondary oxidation products in chilled Argentine sandperch muscle.

Fish and fish products are very prone to discoloration during conservation. The differences in lightness, hue and chroma between the control and treated fish flesh have been shown to be most probably caused by the increased levels of RO in the flesh. Rosemary extract partially prevented the loss of red color in chilled Argentinian sea perch muscle [26].

### 3.2. Fatty Acid Profile

As far as FAs’ composition of treated or untreated flesh is concerned, C16:1, C18:1*n*9 and C18:3*n*3 contents were not influenced by RO treatment, while C18:3*n*3, C20:1, C20:2*n*6, and C20:4*n*6 contents were not statistically different during storage, while the other FAs were affected by antioxidant treatment and by storage time (Table 2). Changes in the composition of the saturated FAs (C14:0, C16:0 and C18:0), mono-unsaturated FAs (C18:1*n*7 and C20:1) and poly-unsaturated FAs (C18:2*n*6, C18:4*n*3, C20:3*n*3, C20:4*n*3, C20:5*n*3, C22:5*n*3 and C22:6*n*3) suggest that all these FAs could be involved in the oxidation process in a different manner. This could be related to the oxidation of unsaturated FAs that lead to a change in the percentage of FAs. The present study showed that RO was able to inhibit the oxidation of C20:5*n*3, C22:5*n*3 and C22:6*n*3, which are the main acids involved in this process.

**Table 2:** Fatty acids (FA) composition (g/100 g total FA) of flesh ranged according to treatment ^a^ and storage time.

	C		RO 0.2		RO 1.0		RO 3.0		SEM	Significance		
Days	3	9	3	9	3	9	3	9		Treatment	Time	Interaction
C14:0	3.76	3.12	3.63	2.41	3.02	2.81	2.50	3.05	0.067	0.003	0.002	0.000
C16:0	20.0	15.6	20.0	11.9	15.2	12.8	14.7	14.7	1.319	0.000	0.000	0.000
C16:1	4.33	3.77	4.23	3.20	4.13	3.37	3.33	4.17	0.127	0.356	0.018	0.002
C18:0	6.17	5.30	6.20	3.87	4.37	3.80	4.40	4.03	0.077	0.000	0.000	0.000
C18:1*n*9	16.9	14.1	17.0	12.3	15.3	11.8	13.2	15.9	1.739	0.179	0.001	0.001
C18:1*n*7	2.90	2.40	2.83	1.93	2.37	2.03	2.07	2.40	0.038	0.004	0.000	0.001
C18:2*n*6	14.0	13.4	15.4	15.3	19.4	9.50	17.1	20.9	2.424	0.000	0.015	0.000
C18:3*n*3	1.27	1.40	2.77	1.83	2.23	0.70	2.07	2.40	0.618	0.100	0.139	0.170
C18:4*n*3	0.00	0.27	0.00	0.12	0.00	0.00	0.32	0.06	0.022	0.001	0.018	0.000
C20:1	0.77	0.87	0.73	0.60	0.33	0.00	0.60	0.63	0.011	0.000	0.068	0.011
C20:2*n*6	0.63	0.60	0.00	0.67	0.73	0.00	0.73	0.80	0.003	0.000	0.710	0.000
C20:3*n*3	1.30	0.50	0.77	1.23	0.37	2.53	0.30	0.40	0.292	0.023	0.043	0.002
C20:4*n*6	0.00	0.00	0.00	0.33	0.70	0.00	0.70	0.70	0.012	0.000	0.054	0.000
C20:4*n*3	0.00	0.00	0.00	0.17	0.73	0.00	0.63	0.70	0.011	0.000	0.011	0.000
C20:5*n*3	2.03	2.57	2.13	3.50	6.33	1.10	6.33	5.90	0.132	0.000	0.000	0.000
C22:5*n*3	0.00	0.00	0.00	1.40	2.23	0.00	2.17	2.30	0.010	0.000	0.000	0.000
C22:6*n*3	5.83	5.63	5.60	8.57	14.93	12.47	17.83	13.97	0.835	0.000	0.000	0.000

^a^ C: control; RO 0.2: rosemary oil 0.2%; RO 1.0: rosemary oil 1.0%; RO 3.0: rosemary oil 3.0%.

Tironi *et al*. [26] reported a protective effect of rosemary extract on the lipid fraction of Argentinian sea perch muscle and demonstrated that lipid oxidation and C22:6*n*3 content modification were prevented by the addition of rosemary extract.

Tironi *et al*. [25] found that rosemary extract (200 mg/kg) was able to inhibit the oxidation of C22:6*n*3. However, this type of antioxidant could not prevent C22:5*n*3 and C20:4*n*6 oxidation. Thus, their oxidation could be responsible for the increasing TBARS values in treated samples after four months of storage.

### 3.3. Biogenic Amines Content

Differences in the concentration of biogenic amines was found to be due to storage time (increasing with time) and antioxidant treatment (decreasing with antioxidant addition) with the exception of spermidine content, that was not influenced by treatment and time (Table 3). The analytical determination of BAs suggested that the rosemary treatment exerts a significant protective effect on the degradation of proteins. In fact, the BA content was reduced proportionally to the RO quantity used. This effect would be an antimicrobial effect, because these BAs are produced through bacterial decarboxylase activity.

**Table 3:** Biogenic amine contents (μg/kg) of flesh ranged according to treatment ^a^ and storage time.

	C		RO 0.2		RO 1.0		RO 3.0		SEM	Significance		
Days	3	9	3	9	3	9	3	9		Treatment	Time	Interaction
Putrescine	27.2	46.5	24.6	37.0	10.7	24.9	4.7	6.5	18.8	0.000	0.000	0.052
Cadaverine	16.9	29.6	15.6	22.2	6.1	18.0	2.2	3.8	35.5	0.007	0.025	0.559
Spermidine	2.1	1.8	1.7	2.4	1.6	1.4	1.1	1.2	0.57	0.293	0.930	0.759
Tyramine	13.4	16.0	17.0	13.5	2.8	14.9	2.8	4.6	6.4	0.000	0.015	0.004
Histamine	17.3	320.1	103.4	271.2	5.0	50.9	8.0	16.5	2808	0.000	0.000	0.001
Spermine	ND	ND	ND	ND	ND	ND	ND	ND	-	-	-	-
Dopamine	ND	ND	ND	ND	ND	ND	ND	ND	-	-	-	-

^a^ C: control; RO 0.2: rosemary oil 0.2%; RO 1.0: rosemary oil 1.0%; RO 3.0: rosemary oil 3.0%; ND, not detected.

There is no reference at all to maximum concentrations of BAs in fish products, with the exception of histamine. The Food and Drug Administration [27] sets the limit of histamine tolerance in fresh fish at 50 mg/kg, while European legislation sets the permitted histamine levels in fishery products from the scombroid or cupleoid family at 100–200 mg/kg for fresh fish, and up to 400 mg/kg for cured products [28].

Özyurt *et al*. [16] reported that icing containing rosemary extract had a positive effect on sardine during chilled storage, causing low BA content, especially histamine and putrescine.

Özogul *et al*. [14], studying the effect of rosemary and sage tea extracts on BA formation in vacuum-packed sardine fillets stored at 3 °C, found that their content generally increased in all treatments with increasing storage time, and that putrescine and cadaverine were the most abundant BAs formed in sardine muscle. 

Özogul and Özogul [29] found that BA content in sardine was highest in sardine stored in air, followed by modified atmosphere pack and vacuum pack. Lower putrescine and cadaverine contents were found for sardine held in vacuum package conditions, while spermidine and spermine levels increased slightly and did not change much throughout the storage period in sardines (*Sardina pilchardus*) stored in modified atmosphere packaging and vacuum packaging. Ababouch *et al*. [30] found slight increases in spermine and spermidine levels in sardines during storage at room temperature.

### 3.4. Characterization and Content of Terpenoid Component

It is also possible to evaluate the presence of the terpenoid fraction of RO in fish flesh: the terpenoid component profile is clearly present in higher amounts in treated samples, even though a small enrichment in less volatile compounds is visible (Table 4). This is probably due to the freeze-dried process of flesh samples, in which highly volatile components were partially lost.

**Table 4:** Terpene and sesquiterpene compositions (arbitrary area units) of flesh ranged according to treatment ^a^ and storage time.

	RO 0.2		RO 1.0		RO 3.0		SEM	Significance		
Days	3	9	3	9	3	9		Treatment	Time	Interaction
α-Pinene	10,609	14,498	16,228	12,228	334,740	555,058	2.1 × 10^9^	0.000	0.005	0.002
Eucalyptol	4366	30,740	133,773	224,546	927,153	1,293,565	3.5 × 10^9^	0.000	0.000	0.001
Camphor	18,831	71,698	158,171	243,992	1,125,807	146,497	3.8 × 10^9^	0.000	0.000	0.000
Borneol	547	3106	13,458	26,446	43,900	38,061	8.4 × 10^6^	0.000	0.036	0.000
Isoborneol	4525	36,386	49,713	66,694	398,769	409,514	1.7 × 10^8^	0.000	0.007	0.382
α-Terpineol	ND	ND	36,132	40,596	391,096	495,470	2.7 × 10^8^	0.000	0.001	0.000
Bornylacetate	ND	5493	2278	1598	69,125	50,527	5.0 × 10^6^	0.000	0.001	0.000
β-Caryophyllene	ND	ND	5332	3084	250,712	176,756	2.2 × 10^8^	0.000	0.004	0.001

^a^ RO 0.2: rosemary oil 0.2%; RO 1.0: rosemary oil 1.0%; RO 3.0: rosemary oil 3.0%; ND, not detected.

## 4. Conclusions

The results indicate that application of RO to MTM may be advantageous. RO improves the pH, oxidative stability of the lipids and FA profile, which resulted in a significant extension of MTM shelf-life. Treatment with RO had a positive effect, leading to low BA content, especially putrescine, cadaverine, tyramine and histamine. Moreover, the presence of the terpenoid fraction of RO in fish flesh improved the quality of this ready-to-cook fish.
